# Around the Hospitals

**Published:** 1893-02-25

**Authors:** 


### AROUND THE HOSPITALS.

**The London Hospital,** the largest of our Metropolitan hospitals, and certainly one of the most usefully situated, has again reached the point in its history when special efforts are necessary on its behalf. Since 1878 the custom of making a special appeal every five years has been established. So far the results have been most excellent. Last week a meeting was held to consider the financial position of the hospital and the advisability of a special appeal after the manner adopted on previous like occasions. The meeting decided that the Lord Mayor should be asked to call a meeting at the Mansion House, to be held in April, in furtherance of this appeal, and that H.R.H. the Duke of Cambridge should be invited to preside. Since the last quinquennial appeal, the hospital has been immensely improved and added to. The management cannot do better than induce all to visit the magnificent hospital for themselves, worthy in every way of the nation and metropolis. No surer method could be devised for awakening public interest. The present adverse balance, we feel sure, would be promptly removed could the wealthy members of our community realize for themselves the vast work of the London Hospital.

**The Seaman's Hospital,** which is accomplishing an ever-increasing amount of work, is unfortunately suffering from want of adequate funds in common with many other charitable institutions. As the Lord Mayor, presiding at the recent annual meeting of the hospital, pointed out, no hospital is so intimately connected with our trade and commerce as this, and Sovereigns of all nations have recognised the benefits it universally confers. Possibly, the wandering nature of those benefited may act prejudicially on the funds, although this should not be the case, having regard to the number of rich employers of the labour of those who form so large a portion of the patients. The branch institutions have proved, by the numbers benefited by them, the necessity for their existence. The need for like assistance at Tilbury is very urgent, but unfortunately money comes in very slowly, and there seems no immediate possibility of carrying out the project, although the dock company have expressed their willingness to take their share in its maintenance. We trust that the large and influential nature of the recent meeting at the Mansion House will be the means of awakening increased interest and affording substantial help to the hospital.

